# Ectopic Thymus in Paediatric Thyroid: Support for Conservative Management

**DOI:** 10.7759/cureus.82323

**Published:** 2025-04-15

**Authors:** Padma Rao, Jason Quick, Patrick A Dewan

**Affiliations:** 1 Radiology, The Royal Children's Hospital, Melbourne, AUS; 2 Radiology, University of Melbourne, Melbourne, AUS; 3 Paediatric Surgery, Sunshine Private Hospital, Melbourne, AUS

**Keywords:** ectopic, elastography, thymus, thyroid, ultrasound

## Abstract

We present a case of conservatively managed intrathyroidal thymic tissue in a 3.5-year-old girl, who had an ultrasound for a midline swelling that was consistent with a dermoid cyst, proven by operative specimen pathology. An incidental finding was that of thymic tissue within the thyroid, the ultrasound characteristics of which enable differentiation from thyroid malignancy, enabling a confident diagnosis of this benign, asymptomatic condition. Thus, rather than proceeding to invasive investigations, detailed ultrasound analysis obviates the need for thyroid biopsy. The features that enable malignancy to be excluded are discussed.

## Introduction

Ultrasound is a first-line investigation used in the assessment of the thyroid gland. Advances in ultrasound technology have enabled both the detection of previously unrecognised abnormalities and improved characterisation of detected lesions. High resolution sonographic evaluation of thyroid abnormalities can greatly assist in differentiating between benign and malignant lesions and is used to guide appropriate management [1].

During embryonic development, the thymus and thyroid are anatomically intimate [2]. During the caudal migration of the thymus, portions of the thymic tail fragment into small pieces creating the thymopharyngeal tract, with thymic remnants being deposited along this tract. Normally, the tract and remnants disappear. Defective embryogenesis and subsequent aberrant migration may result in thymic remnants persisting along the tract between the neck and upper mediastinum or in aberrant locations, including within the thyroid gland [3].

Ectopic intrathyroidal thymic tissue (EITT) is a benign, asymptomatic condition, which is usually detected incidentally when the neck is being evaluated for other reasons [4]. Lack of familiarity with the typical sonographic characteristics of thymic tissue may prompt further investigation to differentiate between benign and malignant pathology.

Historically, EITT investigations include invasive techniques such as fine needle aspiration or lobectomy, the invasive nature of which raises the anxiety and concerns of the patient and their family. However, demonstration of characteristic features of thymic tissue by high resolution ultrasound allows for a conservative pathway to be adopted, namely surveillance US imaging considered necessary for follow up.

The objective of this study was to review the clinical presentation, sonographic findings, management and outcomes for EITT patients recorded in the literature and present a case report of ectopic intrathyroidal thymic rests in a paediatric patient, aiming to identify the pertinent clinical and sonographic features that would allow a confident diagnosis of ectopic thymic tissue, avoiding the need for further invasive investigations.

## Case presentation

A 3.5-year-old girl presented with a two-month history of a midline cervical swelling that was clinically and sonographically consistent with a midline dermoid cyst, which was confirmed histologically when later removed. There was no history of thyroid dysfunction and the patient was otherwise well, apart from a small persistent umbilical hernia.

**Figure 1:**
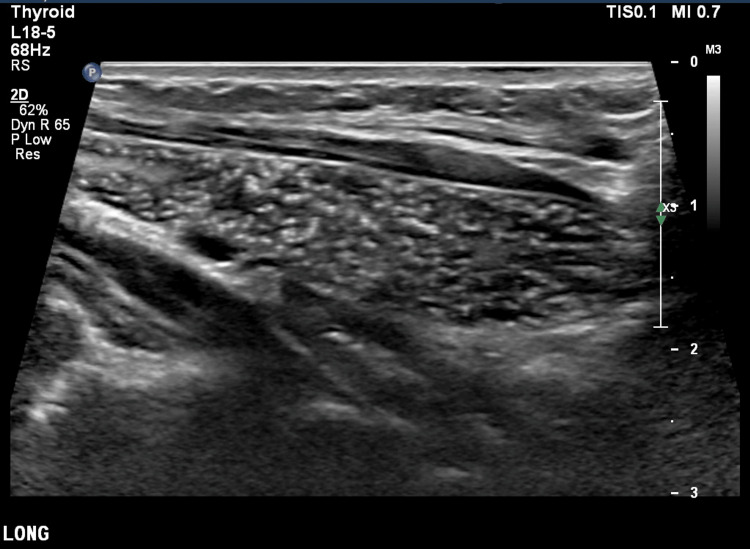
Longitudinal high-resolution ultrasound of the thymus demonstrating the characteristic starry-sky appearance due to multiple linear and punctate echogenic foci

**Figure 2:**
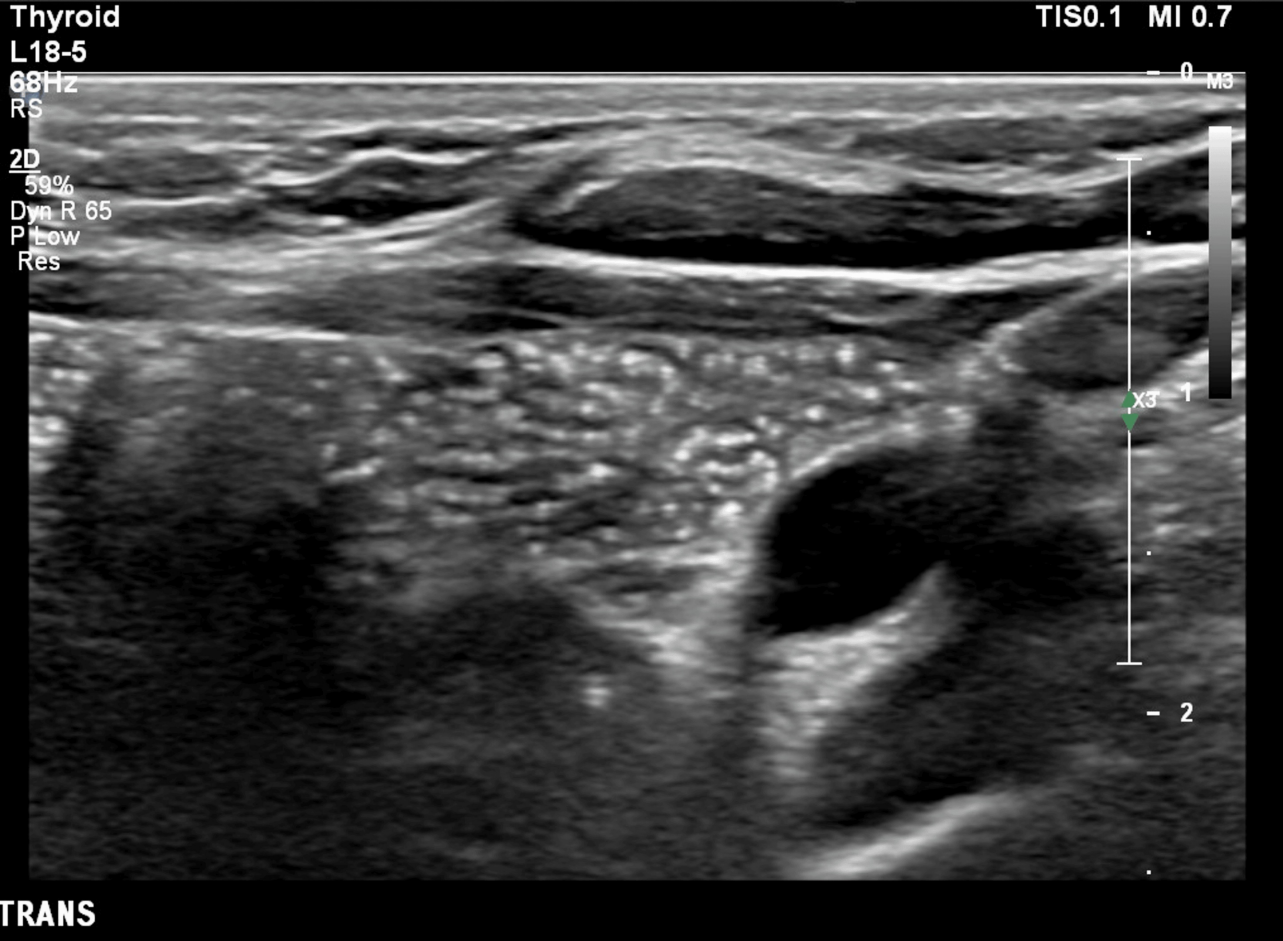
Transverse high-resolution ultrasound of the thymus demonstrating the characteristic starry-sky appearance due to multiple linear and punctate echogenic foci

**Figure 3:**
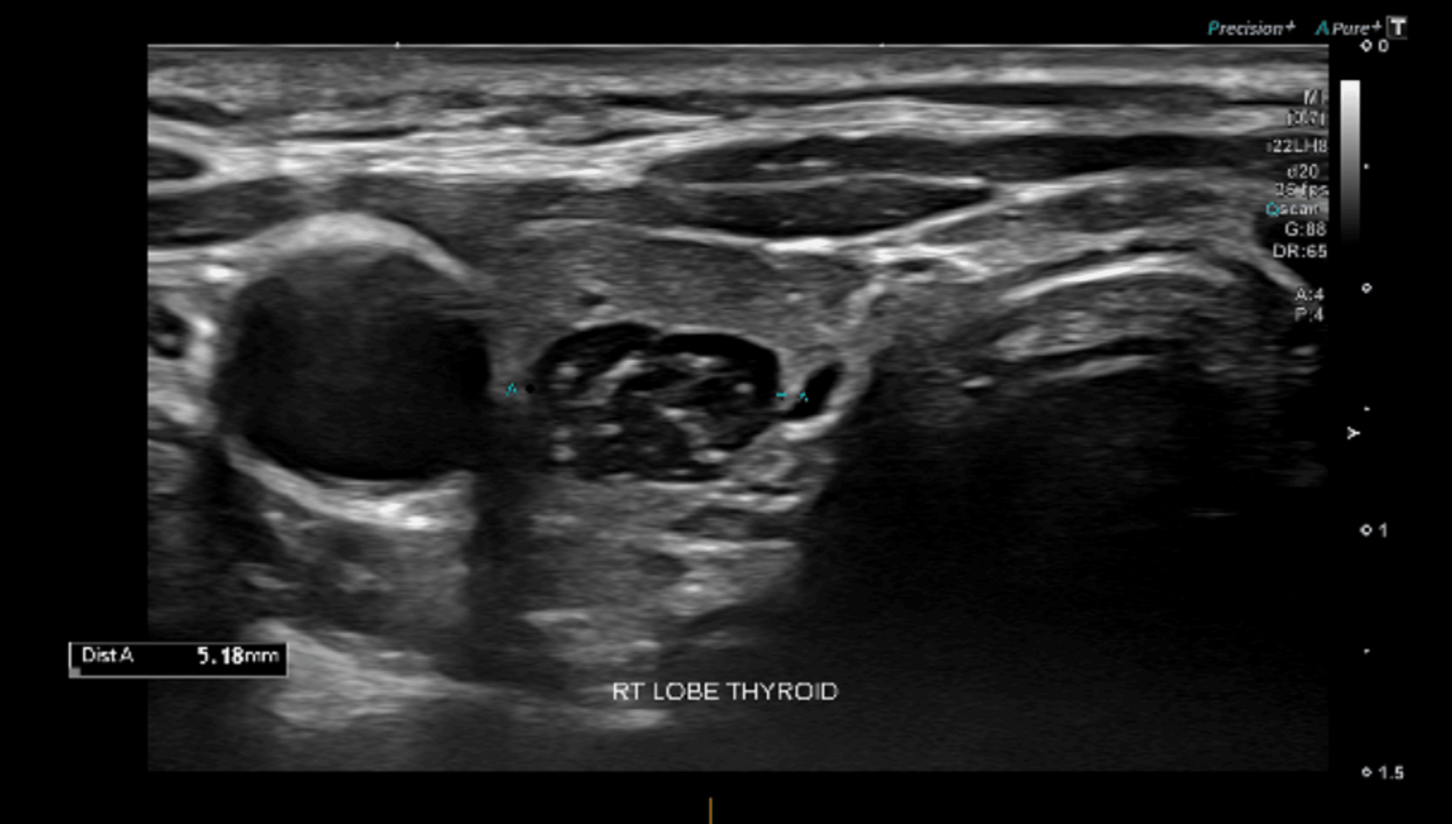
Transverse view of intrathyroidal thymic nodules seen as well demarcated hypoechoic nodules containing linear and punctate echogenic foci typical of thymic tissue

At the time of the ultrasound of her neck, the thyroid gland was noted to be abnormal with bilateral hypoechoic fusiform and longitudinal nodules containing multiple internal echogenic foci, as shown in Figures 1, 2, 3.

The clinical, biochemical and ultrasound findings were reviewed by the radiology, endocrinology, and ear, nose and throat (ENT) surgical teams, with the consensus that the intrathyroidal lesions were typical of ectopic thymic tissue. Thus, conservative management was embarked upon and the patient remained well with stable ultrasound findings on follow up imaging 35 months later, with persistently normal thyroid function.

There was no history of thyroid dysfunction and the patient was otherwise well, apart from a persistent umbilical hernia.

The clinical, biochemical and ultrasound findings were reviewed by the radiology, endocrinology and ENT surgical teams, with the consensus that the intrathyroid lesions were typical of ectopic thymic tissue. Thus, conservative management was embarked upon and the patient remained well with stable ultrasound findings on follow-up imaging 30 months later.

## Discussion

The decision to adopt a conservative approach was made after careful evaluation of the history, clinical examination and review of the biochemical and ultrasound appearances. Identification of the tissue as ectopic thymus requires experience and knowledge of the typical sonographic appearances of thymic tissue and the use of appropriate high-resolution ultrasound probes.

Identifying ectopic thymic tissue characteristics

Thyroid nodules are rare in children and concern may be raised for malignancy as the ultrasound appearances of EITT can resemble those of thyroid papillary carcinoma [5]. The classic hallmark appearance of ectopic thymic tissue is of variably sized, well-demarcated hypoechoic soft tissue mass, often fusiform, longitudinal or ovular in shape, with multiple linear and punctate echoes, giving a ‘salt-and-pepper’ or ‘starry-sky’ appearance [6]. Pathological thyroid nodules are usually rounded [7].

Elastography

Elastography is a non-invasive technique that helps in determining tissue stiffness in various organs and may be useful in confirmative diagnosis [8]. It directs low frequency vibrations into the tissue and ultrasound is used to measure how quickly the vibrations move through the organ. When tissues are affected by pathological processes, the tissue elasticity changes, which is reflected in changes in tissue elastography values. However, EITT and the surrounding healthy thyroid tissue are similar in stiffness. Conversely, malignant nodules differ in stiffness leading to different elastography values [9].

‘Watch-and-wait’ philosophy

Knowledge of the typical ultrasound characteristics of EITT is central to adopting in a watch and wait philosophy when thyroid nodules are identified [10]. In a large, retrospective review by Purcell et al., 115 lesions were ultrasonographically identified as EITT, and only one patient underwent surgery to rule out malignancy, while another underwent surgery due to the onset of compressive symptoms [11]. This supports the shift toward conservative management, with imaging being central to the ongoing decision-making and management process.

Frates et al. propose a surveillance imaging algorithm, encouraging conservative principles with pathways for more invasive investigations based on lesion size, or progression of lesion size [12].

## Conclusions

Although EITT is an uncommon entity, the ultrasound features are characteristic. Therefore, the incidental anomaly can be diagnosed without resorting to biopsy. In particular, if elastography is used to complement conventional ultrasound scan findings, there is increase in the diagnostic certainty.
